# Advances in Musculoskeletal Imaging and Their Applications

**DOI:** 10.3390/jcm12206585

**Published:** 2023-10-18

**Authors:** Adam Piórkowski, Rafał Obuchowicz, Andrzej Urbanik, Michał Strzelecki

**Affiliations:** 1 Department of Biocybernetics and Biomedical Engineering, AGH University of Science and Technology, 30-059 Krakow, Poland; 2 Department of Diagnostic Imaging, Jagiellonian University Medical College, 31-008 Krakow, Poland; rafalobuchowicz@su.krakow.pl (R.O.); aurbanik@mp.pl (A.U.); 3 Institute of Electronics, Lodz University of Technology, 93-590 Lodz, Poland; michal.strzelecki@p.lodz.pl

## 1. Introduction

Modern medical imaging systems provide ever-more information about the patient’s health condition. Precision, resolution, and sensitivity are increasing, and new possibilities of differentiating the condition of tissue appear. Such advances are also taking place in musculoskeletal imaging. Ever-more accurate imaging in various modalities allows us to discover new relationships between the image and the diagnosis. It is, therefore, important to use all this information to best serve the patient, hence research related to the analysis of medical images is so important, because it allows us to indicate what is invisible to the naked eye or quantify what has so far been measured by humans or subject to discretionary assessment.

This collection includes 25 works related to the analysis of images created during diagnostics of the musculoskeletal system using various modalities, starting with X-ray and fluoroscopy, before moving onto conventional or spectral computed tomography, magnetic resonance imaging, ultrasound, and elastography and PET/CT and ending with systems for analyzing patient mechanics. Research was carried out related to the detection of various conditions, parameterization of clinical and population phenomena, and detection of image–clinical condition relationships. Despite the current popularity of machine learning techniques [1], the collection is made up of classic engineering methods related to image processing. Several works use textural analysis, which has not been appreciated so far and is particularly useful in relation to the imaging of the structure, especially the bones.

## 2. Texture Analysis in Musculoskeletal Imaging

Recently, radiomics has played an increasing role in analysis of biomedical images, being a tool for the quantitative description of digital image content. Radiomic features can be divided into histogram-based, texture-based, and shape-based features [2]. Of most interest are texture-based parameters that describe the structure and properties of visualized tissues and organs. Such parameters, if properly selected and repeatable, are very useful for reliable characterization of internal human organs independently of the image modality used. It has been demonstrated that texture analysis can be usefully implemented for classifying median nerves in carpal tunnel syndrome in echo images [3], diagnosis of cysts and granulomas in intra-oral radiographs [4] or quality assessment of MR images [5]. There is also free software available for texture feature evaluation, such as MaZda [6] and its successor qMaZda [7], widely used by many researchers to facilitate texture analysis in many medical image processing tasks. Such an approach is also present in some of the papers included in this Special Issue.

In [8,9], the research question discussed whether corticalization in radiographs is related to a higher risk of bone tissue loss adjacent to dental implants in smoking patients. Dental implant research has frequently delved into the factors that contribute to implant failure due to marginal bone loss. Smoking, known for its detrimental effects on health and bone structure, plays a role in impacting oral health and jawbone condition. The study is aimed at exploring how tobacco smoking influences the peri-implant jawbone’s corticalization. Texture features were analyzed for radiographs, and corticalization around the implant was investigated. MaZda software was employed for this task. The study that covers a 5-year observation of radiograph bone texture established a link between smoking and alterations in the tissue structure near dental implants. The corticalization phenomenon, which can be immediately detected post-implantation, is a crucial marker. It may serve as an early indicator of the implant’s likelihood of success. Understanding the correlation between smoking and resulting changes in bone structure around dental implants allows physicians to make more informed decisions about the viability of implants for smokers versus non-smokers. Physicians can provide tailored advice and counseling to patients who smoke, highlighting dental implants’ potential risks and outcomes. This can potentially lead to better patient compliance and health outcomes.

Research described in [10] aimed to elucidate the reasons behind marginal bone loss (MBL) post-dental implant insertion without functional loading, focusing on understanding bone alterations surrounding the implant neck. A noticeable association between MBL and higher torques was evident after 3 months, albeit exclusively in the mandible. Over time, the radiographic texture features—sum average, entropy, difference entropy, long-run emphasis, short-run emphasis, and discrete wavelet decomposition transform attributes—underwent alterations. The study found that MBL correlates with the torque level applied during the dental implant insertion and the procedure’s anatomical placement. Understanding the relationship between torque during implant insertion and MBL provides physicians with valuable information to optimize their technique and minimize MBL in patients. Using insights from the study, physicians can ensure that patients have the best potential outcomes from dental implant procedures, especially in terms of minimizing bone loss.

The author of [11] investigates the behavior of bone index (BI) values in regions of bone loss characterized by radiographically translucent non-trabecular areas to propose alternative indices specifically designed for detecting corticalization in living bone via the use of textural analyses. The study provides clinicians with valuable insights into the changes that occur in peri-implant bone over time after dental implant insertion. By understanding how bone index (BI) values change and the phenomenon of corticalization, clinicians can make more informed assessments of bone health surrounding dental implants. The study introduces objective measures, such as mean optical density, entropy, and differential entropy, which can help clinicians to quantitatively assess changes in peri-implant bone. These measures provide clinicians with reliable data to accurately track bone changes and monitor the progression of bone remodeling.

The objective of the research presented in [12] was to discern a link between textural characteristics discerned in X-ray skeletal images and the ages of the subjects. Through rigorous visual scrutiny of the images, the study sought correlations between textural attributes and chronological age. The investigation pinpointed five specific anatomical landmarks for analysis on both sides of the body, which were the iliac wing, femoral neck, greater trochanter, ischium, and femoral shaft. Each landmark’s textural attributes were systematically measured. Of all landmarks, the left femoral neck showcased the most pronounced relationships. Specifically, the textural patterns derived from the histogram of oriented gradients and the gray-level co-occurrence matrix were most correlated with age, presenting a correlation coefficient (ρ) of −0.52 and a significant *p*-value of 4.95 × 10^−14^. The primary revelation from this investigation is that age-related structural variations in the femoral neck region are more profound compared to other femoral components. By understanding the correlations between textural features in X-ray images and age, physicians can make more informed diagnoses linked to bone health- and age-related changes. The methodology proposed can be pivotal in enabling the early identification of osteoporosis. Early detection enables timely interventions, which can improve outcomes and reduce the risk of fractures.

Another paper [13] considers the bone age evaluation of adolescent wrists in boys from MRI Images. Such a bone age is typically X-ray assessed. It makes possible the assessment of the child’s development and is an important parameter of the child’s proper growth. However, diagnosing a specific disease is not enough. Diagnoses and prognoses often depend on the degree to which the selected case varies from the bone age norms. The bone age examination could then become a standard screening test. Replacing the technique for the bone age evaluation would also protect the patient against harmful dose of ionizing radiation, which results in a less invasive test. The experiments performed have shown that MRI texture representing wrist region ensures robust results in the determining of bone age while the patient is not exposed to ionizing radiation. Since MRI does not involve ionizing radiation, doctors can recommend it without concerns about exposing their patients, especially younger ones, to potential radiation risks. This finding means that repeat examinations, if necessary, are safer. The potential to normalize bone age evaluations as standard screenings would offer physicians a consistent method to track and evaluate developmental progress.

## 3. Applications of Image Analysis in Musculoskeletal Imaging

For patients with medial open-wedge high tibial osteotomy (MOWHTO), unintended distal tibial rotation is observed [14]. While computed tomography (CT) is conventionally used for lower limb alignment assessments, the novel low-dose EOS system now offers automated three-dimensional limb modeling and alignment measurement. This research juxtaposed alignment modifications, post-MOWHTO, detected through the EOS system and CT. This finding highlights the EOS system’s potential as a viable alternative to CT in evaluating specific pre- and post-surgical parameters. The propensity of the distal tibia to undergo internal rotation post-MOWHTO was verified, albeit without identifying the significant factors contributing to this deformation.

One potential benefit for physicians is that the EOS system, being a low-dose imaging modality, exposes patients to significantly less radiation compared to standard CT scans. This allows physicians to more frequently order imaging if required without subjecting patients to high radiation doses. Three-dimensional limb modeling gives physicians a comprehensive view of the limb, which could be essential for surgical planning and assessment. Automation feature in EOS can potentially save physicians time, as manual measurement can be time-consuming and prone to human error. As the study suggested that there was negligible variation between CT and EOS in terms of capturing pre- and post-operative changes, physicians can feel confident using the EOS system as a reliable diagnostic tool. Lower radiation exposure and potentially shorter imaging time can enhance the patient’s comfort and experience, which indirectly benefits the physician by facilitating easier patient management and improved patient compliance. Depending on the physical setup and equipment requirements, the EOS system might offer benefits in terms of space utilization or ease of operation compared to traditional CT systems. Incorporating advanced imaging technologies like the EOS system can enhance the systems’ diagnostic capabilities, patient care, and operational efficiency for physicians.

Bone marrow edema (BME), indicative of acute fractures, is difficult to discern using traditional computed tomography (CT) scans [15]. The efficacy of the three-material decomposition (TMD) method for identifying traumatic BME in extremities using spectral computed tomography (SCT) has been studied. The bone compartments analyzed included the distal radius, proximal and distal tibia, proximal femur and fibula, and the long bone diaphysis. Two radiologists, uninformed of the BME status, reviewed these cases in a randomized sequence to determine BME presence. Consistency between the two raters was high, with an inter-rater reliability of 0.84 (*p* < 0.001). Individual bone compartments demonstrated sensitivities ranging from 86.7 to 93.8% and specificities ranging from 84.2 to 94.1%. Positive predictive values varied from 82.4 to 94.7%, while negative predictive values were between 87.5 and 93.3%. The proposed TMD method offers robust diagnostic accuracy in detecting BME in extremities, suggesting its potential as a routine diagnostic tool in emergency scenarios.

The TMD method on SCT provides high sensitivity and specificity, enabling physicians to make confident and accurate diagnoses of BME and acute fractures. With a reliable method to detect BME, physicians can quickly determine the best course of treatment for patients, potentially leading to faster recovery times. Given the highlighted potential of this approach in emergency situations, it could be invaluable for physicians in these settings, where time-sensitive decisions are crucial.

T2 mapping has been studied for its capability to detect and quantify anomalies in the long biceps tendon (LBT) [16]. The research aimed to understand how effectively T2 mapping can identify arthroscopically confirmed LBT pathologies and measure T2 values in both healthy and damaged tendons. On the generated T2 maps, precise regions of interest were identified, targeting the sulcal segment of the LBT, to measure average T2 values. Healthy tendons showcased an average T2 value of 23.3 ± 4.6 ms, while tendinopathy-afflicted tendons displayed an increased value of 47.9 ± 7.8 ms. This differentiation yielded a sensitivity and specificity rate of 100% for all diagnostic thresholds between 29.6 and 33.8 ms. T2 mapping is effective in discerning and quantifying healthy and pathological LBTs. This technique can offer insights into the ultra-structural health of tendons, facilitating the timely detection of anomalies.

Physicians can benefit from the study because the clear distinction between healthy and pathological tendons, as demonstrated by the T2 values, allows early and accurate diagnosis of tendon anomalies. With specific T2 values associated with healthy and pathological tendons, physicians can quantitatively track the progress of treatments, whether conservative or surgical.

Hydroxyapatite deposition disease (HADD) is a complex condition marked by the deposition of hydroxyapatite crystals in soft tissues, resulting in inflammation and pain [17]. This review delves into the intricate etiology of HADD, its progression stages, radiological manifestations, differential diagnosis, and treatment options. The role of imaging specialists in its management is also underscored. The evolution of HADD is charted across three distinct phases: pre-calcification, during calcification, and post-calcification. HADD, though intricate, can be effectively diagnosed and managed with a comprehensive understanding of its origins, progression, and imaging characteristics. As imaging plays a central role in both diagnosis and treatment, the expertise of imaging specialists remains invaluable. This review aspires to offer clinicians a holistic perspective on HADD, ensuring optimal patient outcomes.

The review provides a holistic understanding of HADD, ranging from its etiology to treatment, allowing physicians to deepen their knowledge of the disease. By understanding the radiological findings and the potential of HADD to mimic other diseases, physicians can make more accurate diagnoses, reducing the chance of misdiagnosis.

The objective of study [18] was to evaluate the image fidelity of ultra-high-resolution arthrography of the ankle using a photon-counting detector CT. In this experiment, arthrograms were bilaterally captured from four cadaveric samples using both full-dose (10 mGy) and reduced-dose (3 mGy) scanning protocols. Three distinct convolution kernels, characterized by varying spatial frequencies, were employed for the purpose of image reconstruction (ρ50; Br98: 39.0, Br84: 22.6, Br76: 16.5 lp/cm). Optimal osseous tissue representation was observed using the Br98 ultra-sharp kernel (S ≤ 0.043). However, cartilage visualization was enhanced with diminishing modulation transfer functions across dose protocols (*p* ≤ 0.014). Remarkably, the reduced-dose Br76 exhibited a CNR comparable to the full-dose Br84 (*p* > 0.999) and surpassed Br98 (*p* < 0.001) across all tissues. Utilizing a photon-counting detector CT for ankle arthrography with ultra-high-resolution collimation offers remarkable image clarity and detailed tissue analysis, augmenting the inspection of intricate anatomical structures. While osseous structures were best depicted using an ultra-sharp convolution kernel, soft tissues notably benefited from a convolution kernel with a reduced spatial frequency.

The ultra-high-resolution capability ensures that the clinician obtains unparalleled clarity in images, essential for discerning minute anatomical variations or potential pathology. The flexibility to choose between different convolution kernels based on spatial frequencies means that clinicians can optimize image quality based on the specific tissue or structure that they are investigating. The capability of the reduced-dose (3 mGy) Br76 to offer comparable CNR to the full-dose Br84 and even surpass Br98 indicates that clinicians can achieve high-quality images while reducing radiation exposure to the specimen or patient.

Study [19] probed the efficacy of T2 mapping in terms of evaluating the glenoid labrum and distinguishing between intact labral substances and superior labral anterior posterior (SLAP) lesions, utilizing arthroscopy as the benchmark standard. The established mean T2 value for unblemished labral substances was denoted as 20.8 ± 2.4 ms, while it was found to be 37.7 ± 10.63 ms for subjects presenting with SLAP lesions. These findings propose that the methodological assessment and quantification of labral (ultra)structural constitution via T2 mapping may capacitate the differentiation between arthroscopically verified SLAP lesions and a pristine glenoid labrum.

The ability of T2 mapping to distinguish between healthy labral substances and SLAP lesions with high sensitivity and specificity provides physicians with a reliable diagnostic tool. This helps in performing accurate assessment, reducing the risk of misdiagnosis. Before the potential confirmation via arthroscopy, T2 mapping offers a non-invasive method to detect SLAP lesions. This can save patients from unnecessary invasive procedures and related complications.

Shear wave elastography (SWE) is an emergent diagnostic modality employed to discern tissue abnormalities [20]. Within the realm of preventive medicine, the capability of SWE to identify early structural alterations preceding functional deficits offers considerable promise. The study aimed to discern the effect of anthropometric determinants on Achilles tendon rigidity using SWE. Additionally, the influence of diverse sports activities on tendon stiffness was probed to devise preventive medical strategies for elite athletes. Notable gender-based disparities in AT stiffness are evident across distinct sporting disciplines. Sprinters demonstrated the pinnacle of AT rigidity, a crucial aspect to be considered during clinical evaluations. Prospective research endeavors should explore the potential advantages of undertaking musculoskeletal SWE evaluations both before and after sports seasons.

Thanks to performed research, physicians have improved their understanding of how differential stiffness in Achilles tendons based on gender and sport specificity can refine diagnostic accuracy, ensuring that anomalies are not just dismissed as sport- or gender-related norms. By being aware of the normal stiffness ranges for different groups, clinicians can more effectively identify individuals who might be at increased risk of injuries or other tendon pathologies. For interventions such as physiotherapy or surgical procedures, a clear understanding of typical stiffness values can guide treatment modalities and expected outcomes, ensuring optimum patient care.

Study [21] provides insights into the kinematics of the medial gastrocnemius during isometric contractions. This knowledge can be instrumental in understanding muscle performance, adaptations, and potential injury mechanisms, especially for activities that demand a lot from the calf muscles.

The analysis of the differential muscle deformation and force at various ankle angles can guide rehabilitation exercises, strength training, and sport-specific training to optimize performance and reduce injury risks. The identification of increased force generation at dorsiflexion ankle angles due to higher fiber cross-section deformation asymmetry and higher shear strains can guide clinicians in prescribing exercises or interventions, ensuring that the foot’s position is optimized to achieve specific therapeutic goals.

Study [22] aimed to ascertain the potential benefits of CT temporal subtraction (TS) images in enhancing the detection of developing or enlarging ectopic bone lesions among fibrodysplasia ossificans progressiva (FOP) patients. A retrospective analysis of four FOP patients was conducted. By subtracting registered prior CT scans from more recent scans, TS images were generated. Utilizing TS augmented the detection sensitivity for evolving or enlarging ectopic bone lesions in FOP patients for all interpreters.

It was demonstrated that TS provides improved sensitivity in detecting emerging or enlarging ectopic bone lesions, especially in conditions like fibrodysplasia ossificans progressiva (FOP). This can facilitate earlier intervention or treatment adjustments. By leveraging the augmented ability to detect changes over time using TS, physicians can make more informed decisions regarding treatment plans, allowing tailored interventions based on the progression of the condition. By comparing changes over time in one set of images, physicians can quickly identify areas of concern without having to manually compare previous and current scans.

Study [23] was designed to ascertain the optimal pillow choice for individuals with forward head posture (FHP) by analyzing pressure distributions across the head, neck, upper body and the support provided to the spine in various sleeping postures. Participants were assessed using five distinct pillow types: viscose, fiber, cotton, goose feather, and wool. The material composition of a pillow significantly impacted the comfort and support experienced by users, especially in the context of specific spinal alignments, like FHP. Furthermore, an individual’s preferred sleeping orientation influences the efficacy of a pillow material in terms of spinal support and overall comfort.

Physicians can provide evidence-based recommendations on pillow choice to patients presenting with forward head posture (FHP) based on their preferred sleeping position. This can enhance the therapeutic interventions designed for patients with neck or spine issues. Understanding the relationship between pillow material, sleeping position, and spinal alignment enables physicians to tailor advice and treatments for individual patients, optimizing outcomes.

Study [24] sought to discern kinematic variances at the point of initial contact between female futsal athletes with and without antecedent knee injuries through a functional motor pattern assessment. The ancillary objective was to juxtapose kinematic variations between the dominant limb (typically used for kicking) and the non-dominant limb across the entire cohort using the aforementioned test. Athletes devoid of knee injury antecedents demonstrated kinematic profiles more attuned to physiologically optimal stances to circumvent the valgus collapse mechanism, especially in parameters like hip adduction, internal rotation, and pelvic rotation in the dominant limb. Notably, the dominant limb caused increased knee valgus across participants, indicating heightened injury susceptibility for this limb.

The study resulted in the authors understanding the kinematic differences between injured and non-injured female futsal players that physicians must consider. They can identify at-risk players by observing their motor patterns during specific movements. This allows early intervention strategies, possibly preventing further injuries. For players recovering from knee injuries, the findings can guide physiotherapy and rehabilitation interventions, emphasizing the importance of achieving more physiological positions during movement to avoid the valgus collapse mechanism.

Percutaneous plasma disc decompression (PPDD) serves as a minimally invasive intervention strategy for discogenic lumbar pain and symptoms related to disc herniation [25]. Yet, the procedure currently lacks established variables to predict its outcomes. The correlation between the enhancements in epidurographic imagery and procedure success rate was meticulously assessed. Both the Numerical Rating Scale (NRS) and the Oswestry Disability Index (ODI) were employed to gauge pain intensity and functional impairment, respectively, being used preoperatively and one-month post-intervention. Remarkably, groups with improved epidurographic findings displayed significantly superior pain alleviation and procedural success rates compared to those without such enhancements.

Understanding the potential role of epidurography as an outcome predictor can aid clinicians in making better-informed treatment decisions. If a patient demonstrates epidurographic improvement, they may be more likely to benefit from PPDD. Using epidurography as a predictive tool, physicians can better identify which patients are more likely to benefit from the procedure, leading to higher treatment success rates. By recognizing the potential end-point of the PPDD procedure through epidurography, doctors can avoid over- or under-treating, leading to optimized results and reduced patient discomfort or risk of developing a worsened condition.

The diminished thickness of the temporal muscle (TMT) has been identified as an adverse prognostic factor in patients with brain tumors [26]. Separately, chronic subdural hematoma (CSDH) is a neurosurgical condition notorious for its high recurrence and elusive outcome prediction models. The thickness of the temporal muscle could serve as a tangible prognostic marker, potentially aiding in identifying CSDH patients at heightened risk.

The Identification of temporal muscle thickness (TMT) as a potential prognostic indicator can help physicians to quickly stratify patients based on the risk of poor outcomes. Understanding which patients are more vulnerable allows more informed clinical decision-making. Understanding the association between reduced TMT and outcomes such as hematoma volume and post-operative performance can guide surgeons in their approach, post-operative care, and setting realistic expectations for patients and their families.

The primary goal of this research was to scrutinize and juxtapose ultrasonographic findings in the nails and entheses of patients diagnosed with psoriasis and psoriatic arthritis [27]. Disparities were found in the Nail Psoriasis Severity Index scores and the scores derived from the Glasgow Ultrasound Enthesitis Scoring System when comparing patients with psoriasis to those with psoriatic arthritis. Ultrasonographic assessments revealed more pronounced nail abnormalities in individuals with psoriasis, whereas enthesopathic changes in the lower extremities were more prominent in subjects with psoriatic arthritis.

The use of ultrasonography for the examination of nails and entheses can assist in the early detection of abnormalities in patients with psoriasis and psoriatic arthritis. This is vital for making a timely and accurate diagnosis. Understanding the variations in ultrasonographic findings between psoriasis and psoriatic arthritis allows physicians to design more tailored treatment plans based on the specific condition and its severity.

Intraoperative CT-navigation (iCT-navigation) has shown efficacy in enhancing the precision and safety of transpedicular screw insertion during primary spinal operations [28]. Nonetheless, the challenges associated with altered bone structures and fibrotic tissues make revision spinal surgeries complex. The study aimed to assess the fidelity and safety of iCT-navigation during screw insertion at untouched sites compared to previously operated sites in revision thoracolumbar spinal procedures.

The study illustrates that iCT-navigation can enhance the precision of screw placements, even in revision sites, which are traditionally more challenging due to the disrupted anatomy. The utilization of iCT-navigation appears to reduce the risk of complications, as evidenced by the lack of neurological injuries in the patients studied. The iCT-navigation allows the instantaneous identification of unaccepted screws, permitting their immediate adjustment during the same operation, potentially reducing the need for further revision surgeries.

Titanium trabecular cages (TTCs) have been developed as promising implants with the intent to ensure both immediate and long-lasting spinal stability via rapid osseointegration [29]. However, robust radiological and clinical evidence affirming their effectiveness was lacking. The study aimed to assess the reactive bone behavior at plates adjacent to custom-designed TTCs in lumbar interbody operations using positron emission tomography (PET)/computed tomography (CT) with 18F sodium fluoride (18F-NaF). The 18F-NaF PET/CT has been validated as an effective modality to probe the metabolic repair reaction post-TTC implantation, radiographically illustrating the cage’s capacity to incite reparative osteoblastic activity on the vertebral endplate surface.

The study provides clinicians with valuable insights into the reactive bone behavior adjacent to titanium trabecular cages (TTCs) used in lumbar interbody fusion surgeries. This knowledge may aid in better understanding the osseointegration process and inform surgical planning, potentially leading to improved patient outcomes. The use of 18F sodium fluoride (18F-NaF) PET/CT as a tool to assess metabolic–reparative reactions offers clinicians a scientifically rigorous approach to evaluate the success of implantation. This evidence-based assessment can guide clinical decisions related to patient management and treatment adjustments.

Study [30] introduces an innovative and lightweight machine learning-based approach aimed at facilitating the diagnosis of COVID-19 through the analysis of X-ray images. The proposed method offers a rapid and effective means of diagnosing COVID-19 based on medical imaging. The proposed approach has potential as a valuable tool to assist radiologists in enhancing the diagnostic workflow for COVID-19. The method showcased effectiveness and speed in providing accurate diagnoses.

The introduced schema serves as an additional support tool for radiologists in COVID-19 diagnosis. It assists radiologists by offering a structured method for evaluating X-ray images and potentially reducing the workload associated with manual image interpretation. The lightweight nature of the approach allows the quick processing and analysis of X-ray images. This speed can significantly expedite the diagnostic process, enabling doctors to promptly make informed decisions about patient care.

An innovative technique for conducting cervical interlaminar epidural steroid injections (CILESIs) guided via ultrasound (US), which eliminates the reliance on the loss-of-resistance approach for identifying the epidural space, was presented in [31].

The employment of this novel ultrasound-guided technique for cervical interlaminar epidural steroid injections (CILESIs) offers physicians increased accuracy and precision during the procedure. The direct visualization of anatomical structures aids in precise needle placement, minimizing the risk of complications and optimizing treatment outcomes. The use of ultrasound imaging allows physicians to monitor the administration of injectable substances in real time, ensuring that the medication is delivered accurately and without causing inadvertent damage to surrounding tissues. This heightened level of safety can lead to a decrease in adverse events and complications associated with cervical epidural injections.

Antegrade intra-medullary (IM) nailing remains the typical treatment for femoral shaft fractures, yet non-union rates remain high for infra-isthmal femoral shaft fractures based on this approach [32]. Thus, a retrospective case–control study aimed at identifying perioperative radiographic factors linked to non-union in these cases after antegrade IM nailing was performed.

The results of this retrospective case–control study provide physicians with valuable insights into the radiographic risk factors associated with non-union after antegrade intra-medullary (IM) nailing for infra-isthmal femoral shaft fractures. Armed with this knowledge, physicians can make more informed treatment decisions by considering these risk factors and tailoring their approach to reduce the likelihood of non-union. By understanding the radiographic elements that contribute to non-union, physicians can adopt strategies to mitigate these risks during the treatment process. This proactive approach can lead to improved patient outcomes, reduced complications, and enhanced recovery rates, thereby bolstering the physician’s reputation and patient satisfaction.

## 4. Conclusions

We hope that all readers will appreciate this Special Issue and that the collection of the articles will be useful in a clinical or scientific way, as well as inspire further investigations into the domain of musculoskeletal imaging. We would also like to encourage doctors to use various quantitative image processing and analysis methods and expand cooperation with computer scientists and biomedical engineers in this area. New information obtained thanks to such analyses will improve the efficiency and repeatability of the diagnostic process.
